# Moral insanity and psychological disorder: the hybrid roots of psychiatry

**DOI:** 10.1177/0957154X17702316

**Published:** 2017-04-10

**Authors:** David W Jones

**Affiliations:** The Open University, UK

**Keywords:** England, France, insanity, legal defence, M’Naghten, monomania, moral insanity

## Abstract

This paper traces the significance of the diagnosis of ‘moral insanity’ (and the related diagnoses of ‘monomania’ and ‘*manie sans délire*’) to the development of psychiatry as a profession in the nineteenth century. The pioneers of psychiatric thought were motivated to explore such diagnoses because they promised public recognition in the high status surroundings of the criminal court. Some success was achieved in presenting a form of expertise that centred on the ability of the experts to detect quite subtle, ‘psychological’ forms of dangerous madness within the minds of offenders in France and more extensively in England. Significant backlash in the press against these new ideas pushed the profession away from such psychological exploration and back towards its medical roots that located criminal insanity simply within the organic constitution of its sufferers.

## Introduction

It is perhaps now merely a commonplace observation to suggest that the evolution of psychiatry has been influenced by the demands of society to do something about ‘mental disorder’ and that, for many centuries, there has been strong state-driven concern with the control of forms of deviance that might threaten social order (Foucault, 1967; Scull, 1979a). Thus, the capacity to speak authoritatively on forms of mental disorder that might be associated with crime has been an important spur to the development of the profession (Foucault, 2003). Ancient records of criminal justice suggest that the perception of insanity accorded a perpetrator of crime a certain amount of protection from the full weight of punishment (Bracton, *c*.1250; Porter, 2002; Walker, 1968). For much of that time such a perpetrator would have to be understood, in the words of the seventeenth-century jurist Matthew Hale, to be ‘totally deprived of the use of reason’ (Hale, 1736: 31) and such a state would be manifest in very obvious ways such that no expert confirmation of its presence was required (Loughnan, 2007, 2012). A significant shift in thinking occurred around the beginning of the nineteenth century, as a group of self-styled experts in insanity arose from the various branches of the medical profession. As is well documented, the emergence of ‘psychiatry’ occurred in the early decades of the nineteenth century (Marková and Berrios, 2012), formally establishing itself in the 1840s, coalescing around the work of a transnational group of medics mainly based in France, Britain, Germany and the USA (Goldstein, 1987; Hansen, 1998; Richards, 1998; Scull, 1979a). Until this point, much of their work had been of low status, as ‘mad-doctors’ who treated insanity as keepers of the reviled ‘madhouses’ (Boime, 1991). Through the early decades of the nineteenth century, new ambition had emerged to forge a more positive professional identity. While in many respects psychiatry developed as a branch of medicine with roots in biological methods of enquiry, two notable fronts were opened up in the battle to establish the profession, and they both relied on a move away from this paradigm and instead towards a relatively more psychological theorization of the nature of insanity. First, the case was made that ‘moral treatment’ represented a viable form of therapy within the asylums. Second, there was a determination to demonstrate the capacity to provide expert judgements on the existence of forms of insanity that might specifically manifest as acts of criminality. The latter encouraged the theorization and use of such categories of ‘moral insanity’, homicidal and affective monomania.

The prominence of the word ‘moral’, not only as a model of treatment but also as a form of insanity, requires some explanation. At this time, the word had a multiplicity of meanings, referring to a psychological and affective domain of experience as well as indicating an ethical assessment (Rimke and Hunt, 2002). The significance of the doctrine of ‘moral treatment’ as a justification for the construction of asylums, and thus to the new profession of psychiatry, has been relatively well documented (Bynum, 1964; Castel, 1988; Foucault, 1967; Scull, 1979a). Moral treatment was given institutional support through the widespread enthusiasm of governments, particularly in France and Britain, to build asylums from around the middle of the nineteenth century. Moral treatment was, as Foucault observed, a noteworthy step in the introduction of ‘medicine of the mind’ (Foucault, 1967: 274–5). Gone was the reliance on physical treatments that assumed that a balance of humours might be restored by a series of bleedings and purges, and now there was new scepticism towards the administration of restraint or physical ‘punishment’ as means of returning the patient to reason, rather in the manner in which a beast might be trained (Scull, 1979b). Instead, it was assumed that the provision of a pleasant environment and respectful communication would allow patients to take back full control of their own minds (Pinel, 1806; Tuke, 1813). The underlying, and in some ways remarkable, premises were that there was ‘a mind’ constituted of different parts and that, so long as communication could be made with a sane part of the mind, then sanity could re-assert itself. The idea, rooted in ‘faculty psychology’, that the mind could be differentiated fuelled thinking about forms of criminal and moral insanity (Berrios, 1993: 19).

The role played by moral insanity, and the associated family of diagnoses, in the formation of the profession is less well acknowledged than that of moral treatment. The dream of standing tall in the courts as experts in criminal insanity was very alluring to the gentlemen medics who sought a way out of the mire of dismal and stigmatized work in ‘the madhouses’. The notions of ‘moral insanity’, ‘homicidal monomania’ and ‘affective monomania’ offered considerable scope for the claim to high status professional expertise in the detection of criminal insanity. Here were ‘hidden’ disorders whose existence had implications for public safety. They were not available to the scrutiny of naive observation by lay witnesses, jurors or courts; but they could be detected by newly established experts in insanity. The related concepts of moral insanity, monomania and partial insanity emerged largely in France and Britain and grew around the notion that there were forms of insanity that were not marked simply by the loss of reason but were distinguished by the impact on the feelings or morals of an individual.

This paper consists of four sections. First, the theory of ‘moral insanity’, its emergence, and its place and significance within the developing profession of psychiatry in the first half of the nineteenth century will be discussed. Second, the celebrated trials of Edward Oxford (1840) and Daniel M’Naghten (1843) at the Old Bailey in London will be used to emphasize how notions of ‘moral insanity’ were, at this point, distinctly psychological, in terms of both ontology and detection. The latter trial was to be a high water mark for the role of ‘moral insanity’ in the courts as, despite the triumph in the courtroom, the public reaction to such new ideas was less favourable. The third section will describe the arrest and trial of George Victor Townley for the murder of his former fiancé in 1863; this case was to have, through a hostile public response, a decisive influence on the direction of psychiatry in the later decades of the nineteenth century. As described in the fourth section of the paper, psychiatry moved back towards reliance on biological formulations of insanity, including in relation to matters of criminality.

## Moral insanity and the psychological turn

The diagnosis of ‘moral insanity’ itself can be credited to the work of James Cowle Prichard, first in a brief article in *The Cyclopædia of Practical Medicine* (Prichard, 1833: 11) but then more substantially in his *A Treatise on Insanity* published in 1835. His work needs to be understood as emerging from the influence of a wide transnational group of medics who were in the midst of developing what has become the profession of psychiatry. He directly acknowledged the work of the early French school of *aliénistes* dominated by Philipe Pinel and then his pupil Jean-Étienne Esquirol, but he was also crucially influenced by work in German medicine at this time (Augstein, 1996). This period witnessed the formal establishment of the profession of psychiatry in the 1840s, marked by a number of parallel events in Germany, France, Britain and the USA, as professional associations were initiated and specialist journals founded. In Germany, for example, the inaugural meeting of the German Association for Psychiatry and Psychotherapy (DGPPN) took place in 1842, while the Association of Medical Superintendents of American Institutions for the Insane (AMSAII) was formerly set up 1844 in Philadelphia, USA. The French national system of asylums was organized by legislation in 1838, and the journal *Annales Médico-Psychologiques* was first published in 1843. In Britain, the Association of Medical Officers of Asylums and Hospitals for the Insane was founded in 1841,^1^ and it began to publish its own *Asylum Journal* in 1853. The contents of the first edition of this journal, which included a ‘prospectus’ for the future of the new profession, is noteworthy for the presentation of the profession as one with expertise in psychological matters and for the prominence of the claims being made for expertise in criminality. The assertions of psychological expertise appear through the importance accorded to ‘moral treatment’ in the asylums, as advocated by those such as Phillipe Pinel and John Connolly (already a leading figure in the new profession in Britain), and through the prominence given to the work of Ernst von Feuchtersleben. As Professor of Medicine in Vienna (Burns, 1954), he advocated a ‘psychical mode of cure’ (Anon., 1853; see also von Feuchtersleben, 1847), with Parkin (1975) going so far as to note the links to Freud’s work on the significance of dreams and ‘dormant consciousness’. The claim for criminal expertise was evident in the space given in this first edition to a paper by Dr Delasiauve (based at the Bicêtre in Paris): ‘On monomania, in a psychological and legal point of view.’ It emphasized the existence of ‘monomania’ – or ‘emotional madness’ (Delasiauve, 1853: 9), and evidently represented the views of the French school’s significant work in developing the concept of monomania as a psychological issue with considerable relevance to matters of criminal justice.

The root of this ‘French’ criminological concept of monomania can be traced to the work of Pinel, who made the remarkable distinction between forms of insanity through his identification of *manie sans délire* (or mania with delirium, or delusion; Werlinder, 1978) versus *manie avec délire* in his *Traité médico-philosophique sur l’aliénation mentale ou la manie* published in 1801; this book was soon translated into English (Pinel, 1806). An individual in the grip of *manie sans délire* might not show any ‘change in the functions of the understanding’ but instead would suffer ‘perversion of the active faculties, marked by sanguinary fury, with a blind propensity to acts of violence’ (Pinel, 1806: 151). The emergent French school of alienists, eventually led by Pinel’s pupil Esquirol, went on to develop the notion that there were individuals whose heinous acts of violence were driven by some hidden flawed belief or impulse, but who otherwise betrayed no outward sign of abnormality (Goldstein, 1998). Esquirol’s own work on monomania, originally developed in his article for the *Dictionnaire des sciences médicales* (Esquirol, 1819), gave energy to this line of thought, which was to become central to the endeavours of the Esquirol school’s efforts to establish public legitimacy ‘by carving a place for expert psychiatric testimony in the courts of law’ (Goldstein, 1998: 389). Monomania, characterized as a hidden mental disorder, was well placed to take on this role as it required the expertise of the trained medical practitioner to detect it. This undoubtedly helped to raise the profile of psychiatry in France, and the idea of monomania gained a place in French literary circles from the 1820s (Boime, 1991). It also took on a significant life in British fictional literature (During, 1988; Jones, 2016), but more notably the concept was endorsed in English medical journals and, most significantly, it appeared in a number of well publicized criminal trials, as will be discussed shortly. However, Prichard, in defining moral insanity, was to build considerably on Pinel’s understanding of *manie sans délire*, as he argued that ‘moral insanity’ could be detected in a wide variety of people and did not necessarily bear any relationship to violence. He also departed from Esquirol’s views on monomanias, as he saw the derangement as affecting the character of the sufferer rather than as a hidden specific form of insanity. In a famous passage, Prichard described ‘moral insanity’ as a ‘form of mental derangement’ which, like Pinel’s *manie sans délire*, left the ‘intellectual faculties’ intact while the ‘moral and active principles of the mind’ were ‘strangely perverted and depraved’ and:

the power of self government is lost or greatly impaired; and the individual is found to be incapable, not of talking or reasoning upon any subject proposed to him, for this he will often do with great shrewdness and volubility, but of conducting himself with decency and propriety in the business of life. His wishes and inclinations, his attachments, his likings and dislikings have all undergone a morbid change, and this change appears to be the originating cause, or to lie at the foundation of any disturbance … (Prichard, 1835: 4)

In shifting attention from the inevitably violent and towards more everyday types of improper behaviour, Prichard was influenced by important elements of German thinking that took a more holistic view of the relationship between the mind, body and insanity meaning that insanity had to be understood as affecting the whole *character* of the individual (Augstein, 1996; Hansen, 1998). Thus, in important respects Prichard’s definition was very different from that of the French school which had fashioned the concepts of monomania and was powerfully influenced by traditions of ‘faculty psychology’ (Berrios, 1993). The important common link, however, was that here were forms of insanity which could leave the everyday reason of an individual alone, but would severely impede the capacity of an afflicted individual to avoid severely antisocial or downright violent behaviour. This substantial idea was to be used successfully in a number of high profile cases in the 1840s, just as the profession of psychiatry was formalizing its existence.

## Moral insanity in the courts

It is important to note that, while the terms ‘moral insanity’ and ‘monomania’ were used in courts, it was not in the theoretically precise ways propounded in the medical treatises. However, the successful use of such theories, blurred though they may have been, was revolutionary in courts of law. Up to this point in history, the dominant view of insanity was that, in order to be accepted as a defence in court, it would have to deprive the offender of all reason, such that they would be in the condition of a brute when they committed the offence (e.g. Bracton, *c*.1250). The idea that there might be individuals who were fully aware of their surroundings, planned their actions and managed the affairs of their lives, but who were otherwise suffering from a form of insanity that explained their violent behaviour, was largely ushered into the courts by the new experts. It should be acknowledged that by the nineteenth century there was already considerable interest in the exploration of ‘the mind’ as a complex entity (Rousseau, 1969), and indeed there were already signs that, at the Old Bailey in London at least, courts were starting to consider forms of insanity that did not fulfil the criteria of an absence of reason (Jones, 2016). But the nineteenth century witnessed the march of the medical witness, including those who professed expertise on insanity (Eigen and Andoli, 1986; Smith, 1981). As early as 1800, the trial of James Hadfield had set some precedent for the acceptance in court of relatively subtle kinds of madness that did not remove the capacity of individuals to manage their lives and plan their actions (Eigen, 1991). Hadfield’s foiled attempt to assassinate King George III at the Drury Lane Theatre meant he faced the charge of treason (Anon., 1800; Jones, 2016: 62–7; Moran, 1985; Walker, 1968: 74–9); he thus benefited from the safeguards against the actions of unfettered state power and was allowed to employ a defence lawyer. He chose Thomas Erskine, one of the great lawyers of his day, who planned the defence strategy with care and summoned many witnesses who together rebutted the prosecution case that Hadfield was sane, as he had evidently planned the assassination and was fully aware of what he had done. Erskine first argued that the definition of insanity which allowed no element of reason at all was impractical; second, he introduced the concept of ‘delusion’: a part of Hadfield’s mind was suffering from the mistaken belief that he should kill the king. Medical experts, testifying that Hadfield’s battle injuries would have affected his mental functioning, were joined by many witnesses from different parts of Hadfield’s life who were all happy to support the view that he was indeed eccentric to the point of insanity (Jones, 2016). Under the deluge of this evidence and argument, the Judge, Lord Kenyon, interceded and suggested that since the evidence all pointed one way the trial itself had become a foregone conclusion. The prosecution agreed, and the verdict of not guilty was returned. This case was to have long-lasting significance as it ushered in legislation allowing for the special verdict of ‘not guilty on the grounds of insanity’ alongside the stipulation that the party be detained until ‘His Majesty’s Pleasure be Known’ (Moran, 1985: 513).

In France, the dramatic case of Henriette Cornier in 1825 drew attention to the possibilities of this kind of diagnosis (During, 1988). The highly public dispute over the 26-year-old’s seemingly motiveless decapitation of a 19-month-old girl of her acquaintance featured the leading figures of the newly emerging profession (see e.g. Georget, 1826). The eventual commutation of the sentence from execution to lifetime imprisonment with hard labour was greeted critically in the British medical press, as it was thought that she should not have been found guilty at all (Anon., 1827). Indeed the courts in Britain witnessed the highest levels of respect being shown to these new diagnoses. The most startling cases were those of Edward Oxford and Daniel M’Naghten held in 1840 and 1843, respectively, at the Old Bailey in London.^2^ The 18-year-old Edward Oxford had fired pistols at the young Queen Victoria as she rode in her carriage near Hyde Park. His defence against the charge of treason, which carried the death penalty, was insanity, and much the greater part of his two-day trial was concerned with discussion of his sanity. A number of the luminaries of the new emerging profession were in court as expert witnesses, including Drs Connolly, Hodgkin, Chowne and Ferdinand Clarke. The nub of the experts’ case was that the wanton and motiveless act was itself highly suggestive of insanity. Perhaps the central piece of expert evidence was provided by John Connolly (Scull, 1984), whose evidence was, remarkably, entirely based on an interview he had with Oxford in Newgate prison. Connolly’s evidence alluded to Prichard’s definition of moral insanity, as Oxford showed an ‘insensibility as regards the affections’ and no capacity ‘to comprehend moral obligations’, and he summarized ‘the case’ as follows:

… an occasional appearance of acuteness, but a total inability to reason – a singular insensibility as regards the affections – an apparent incapacity to comprehend moral obligations, to distinguish right from wrong – an absolute insensibility to the heinousness of his offence, and to the peril of his situation – a total indifference to the issue of the trial; acquittal will give him no particular pleasure, and he seems unable to comprehend the alternative of his condemnation and execution; his offence, like that of other imbeciles who set fire to buildings, &c, without motive, except a vague pleasure in mischief – appears unable to conceive anything of future responsibility.^3^

The acceptance of Connolly’s expert evidence, based substantially on an interview, was arguably an important breakthrough for the claim for psychological expertise. Additional medical evidence came from Dr Chowne of Charing Cross Hospital who was introduced as a lecturer ‘on medical jurisprudence’. He argued directly that such ‘a propensity to commit acts without an apparent or adequate motive under such circumstances is recognized as a particular species of insanity’. This has, he went to say, ‘been called moral insanity’.^4^ Put alongside the testimony of witnesses to Oxford’s eccentricity, the court accepted the weight of the arguments, and the special verdict of ‘Not guilty, being insane. To be retained at her Majesty’s pleasure’ was returned.

In many ways, the trial of Daniel M’Naghten in 1843 followed a similar course. M’Naghten had also, in broad daylight, attempted the assassination of a leading member of the establishment: in this case the Prime Minister, Robert Peel. While his attempt on Peel’s life was unsuccessful, M’Naghten fatally shot Peel’s secretary, Edward Drummond, and thus found himself on trial for murder at the Old Bailey. This was a trial of huge public interest; the evidence of the new profession was to take centre stage, and the idea that insanity could be detected through interview was to come under some scrutiny. The prosecution case was that M’Naghten had planned his actions: not only had he obtained the weapons and targeted his victim, he had otherwise been running the affairs of his life (paying his rent, working as a wood turner and invoicing his clients). This was all presented as evidence of a sane and organized mind. The defence case, like that of Oxford, was supported by some of the leading figures in British psychiatry. It is now very clear that the medical witnesses were keen to establish themselves as experts in the exploration of the mind. These were doctors who did not merely have knowledge of the physical body, its faults and lesions, but they had the knowledge and skills to detect insanity hidden away in the mind. The tool of investigation was that of the interview: the careful questioning and listening that could detect insanity. A group of doctors had visited M’Naghten in Newgate as he awaited trial. The delegation appears to have been led by Edward Thomas Munro, the last of four generations of the Munro family who had overseen medical matters at Bethlem Royal Hospital.^5^ Munro was entirely clear that he could detect insanity (in this case the presence of delusion) through the interview alone, and he did not need knowledge of past history or any other evidence:

I believe I am able to discriminate between a case where a man is labouring under delusion, and where a man feigns delusion – I am quite satisfied that the prisoner entertained the delusions he was giving utterance to – I have not the slightest shadow of a doubt on the subject – if I had heard nothing of his past history, nor the evidence given to-day, my examination in the prison would certainly have led me to the conclusion that he was insane – coupling that with the history of the two last years of his life, I have not the remotest doubt of his insanity – I am quite satisfied of it.^6^

The defence examined Munro and emphasized this remarkable claim that he was able to detect a disease of the mind through questioning alone: ‘*Q*. Do you mean that you are capable of distinguishing a delusion of mind by questioning the party, that you can satisfy yourself, by going into a cell where a prisoner is, whether his mind is diseased at all?’ Munro’s answer was clear in affirming this point, and he went further in distancing himself from the need to make a physical examination. He was able to use questions to know what was ‘in his mind’: ‘I believe I can, without knowing his previous history – in a great many instances I can, by ascertaining what is passing in his mind … . I think I can ascertain whether a man is really labouring under delusion, by merely questioning him, by questioning him sufficiently.’ Munro went on to make it clear that he was not relying on physical examination of the body as, although ‘there are often appearances about the body’,

I did not feel the prisoner’s pulse, and I purposely abstained, because I all along wished he should not know I was a physician – I believe he did not know any of us were physicians – I thought there was a very wild expression about his eyes, a peculiar expression, but I do not lay much stress on that …

Monro was questioned by the defence as to the unusual nature of the form of insanity that he had detected:

*Q.* Is it now an established principle in the pathology of insanity that there may exist a partial delusion sufficient to overcome a man’s moral sense and self-control, and render him irresponsible for his actions, exciting a partial insanity only, although the rest of the faculties of the mind may remain in all their ordinary state of operation?

Monro’s response makes reference not only to monomania, but also perhaps to Prichard’s description of moral insanity that would leave only an individual’s moral sense affected:

*A.* Yes, it is quite recognised – the distinction between monomania and general mania is quite recognised – I apprehend that monomania can exist distinct from general mania – it can sometimes unquestionably exist to the extent of overcoming a man’s self control – I have no doubt that this partial insanity may exist, and the faculty that it affects may be impaired and destroyed, and yet the monomaniac exhibit all the appearance of sanity, in all other respects – the acutest reasoners on many points, good arithmeticians, good artists, and good architects – I have known great ability on those points, co-exist with disease in others – I have heard the evidence on the part of the prosecution as to his pecuniary transactions, and heard the letter read which answered the advertisement – that does not at all impair my conviction as to his insanity – I have known many lunatics keep accounts with great accuracy – persons affected on one point, where their intelligence is clear on others – it is quite manifest that such persons carry out their designs, with great ingenuity and contrivance; and afterwards, when they have done the act, they are very frequently alive to the consequences of it – they have shown great cunning in endeavouring to escape from the consequences – I have observed it every day.^7^

William Hutchinson, Esq., M.D., who introduced himself as ‘physician’ at the Royal Lunatic Asylum at Glasgow, was one of the group who visited M’Naghten in Newgate. Hutchinson had also ‘examined him by means of questions put to him’, and reported: ‘I found that he was labouring under morbid delusion of the mind – I was perfectly satisfied that those delusions were really felt by him – in my opinion those delusions were quite sufficient to account for the act with which he now stands charged.’ According to the OBP, the final expert was Dr Forbes Winslow, who, after attending the trial and listening to the evidence, said that he had ‘not the least doubt of the existence of the prisoner’s insanity’. Winslow’s evidence was consistent with his recently published book that identified forms of insanity which promoted ‘a morbid desire to sacrifice human life’ despite the fact that ‘no intellectual delusion is perceptible’ (Winslow, 1843: 60). The defence case was summed up (at some length) by the lawyer Alexander Cockburn, who concluded:

I trust that I have satisfied you by these authorities that the disease of partial insanity can exist and that it can lead to a partial or total aberration of the moral senses and affections; which may render the wretched patient incapable of resisting the delusion, and lead him to commit crimes for which morally he cannot be held responsible. (Quoted in Walker, 1968: 94)

The jury, after brief direction from the judge indicating that the medical evidence all pointed in one direction and ought to be taken seriously, returned the verdict of ‘not guilty, being insane’.^8^

The apparent triumph of the M’Naghten trial was to be short-lived, and the verdict in this highly publicized trial provoked a public storm of disapproval. The leader comment in the *The Standard* was typical in its tone:

On Saturday, indeed, the whole process of a criminal trial appeared to have been inverted. The mad doctors, who attended in the modest character of witnesses, were really the persons who charged the Court and the jury, laying down the law of moral responsibility to both, and the judge it was who returned the verdict, under the direction of the mad doctors . . . . If the mad doctor’s evidence upon the existence and degree of insanity is to be received with suspicion, we respectfully submit that upon the question of responsibility, his evidence is not received at all. That is a question for the law, and the law was clear until the *verdict* of Saturday.^9^

Queen Victoria herself let her displeasure be known by writing to the Prime Minister, Robert Peel:

We have seen the trials of Oxford and MacNauhgten conducted by the ablest lawyers of the day – and they allow and advise the Jury to pronounce the verdict of not guilty on account of insanity. Whilst everybody is morally convinced that both malefectors were perfectly conscious and aware of what they did. (Quoted in Walker, 1968: 188)

The Queen seems to have been part of the fashionable reaction to the verdicts, as shown in the press. *The Morning Post* continued the assault by denouncing ‘the quacks’ of ‘the madhouses’ and arguing that the whole notion of ‘partial madness (considering madness as an active physical disease)’ was impossible:

… Since the creation, there has not been an instance of it, whatever the quack keepers of madhouses, who, of course, try to exalt their craft, may say upon the subject; and even supposing such as thing as partial madness to exist in M’Naughten’s case, what connection was there between the surmised partial madness and the murderous act of the villain.^10^

*The Times* on 6 March 1843 used a more sarcastic tone to ask ‘in a spirit of humble and honest earnestness, of hesitating and admiring uncertainty, and of almost painful dubitation’, whether ‘those learned and philosophic gentlemen’ could ‘deﬁne, for the ediﬁcation of common-place people like ourselves, where sanity ends and madness begins, and what are the outward and palpable signs of the one or the other …’ (quoted in Walker, 1968: 95). This public uproar encouraged the government to request a review of the insanity defence, which was instigated within the House of Lords. This led to a series of questions being asked of a panel of judges on how issues of insanity should be handled in court. The answer to these questions about how jurors should consider the state of mind of the accused came to be regarded as ‘the M’Naghten rules’. They suggest that in order ‘to establish a defence on the ground of insanity’,

it must be clearly proved that, at the time of the committing of the act, the party accused was labouring under such a defect of reason, from disease of the mind, as not to know the nature and quality of the act he was doing; or, if he did know it, that he did not know he was doing what was wrong. (Walker, 1968: 100)

Subsequent use of the rules in courts confirmed that this represented a re-assertion of the narrow criteria of the insanity defence. To be defended on such grounds, an individual would have to rebuff any prosecution which might seek to demonstrate that the accused knew what they were doing at the time of the offence and knew it to be wrong. Individuals such as M’Naghten and Oxford, who planned their actions and made no secret of their intentions, would have been doomed to the gallows according to these rules, which reaffirmed the primacy of reason. It seemed, however, to take a little while for the implications of the rules to be fully grasped by the new profession. The claims for courtroom expertise in deciphering hidden forms of insanity remained important, even as the profession established itself on a formal footing through the 1840s and 1850s. However, a case in 1863 removed all doubt, and it encouraged the new profession to retreat from further investigation of the content of the minds of offenders and move instead towards the classification of whole groups of people who made up the so-called ‘criminal classes’, according to biological propensity.

## The Townley affair

George Victor Townley became notorious at the age of 25 when he stabbed to death his former fiancée, Elizabeth Goodwin, in 1863. The 22-year-old gentlewoman had broken off their engagement some days earlier. Apparently distraught, Townley made his way to where she had been staying with her grandfather in Wigwell Hall in Derbyshire. During a walk with Elizabeth, Townley used a knife to stab her deeply in the neck with sufficient ferocity to rupture her carotid veins and arteries. Such violence among members of the gentry might have been reported in the press, but Townley’s subsequent behaviour guaranteed this story would achieve similar levels of publicity to the M’Naghten case (Walker, 1968: 208). Townley did not try to run away, but instead he stayed with ‘Betsie’ as she died. He helped to carry her back to the house, stopping to kiss her and making efforts to stem the flow of blood. Townley thoroughly condemned himself by telling all those around that he had killed her and would do so again. The only possible defence at his trial at Derby on the 11 and 12 December 1863 was that he was insane. The medical man who took on the duty of establishing his innocence was Dr Forbes Winslow. He based his case on two separate interviews with Townley and on his prior experience. He was, by this time, an arguably well established expert in the field of ‘criminal insanity’. In November, two months after the killing, Winslow interviewed Townley for nearly two hours, and then for three-quarters of an hour the day before the trial.

Winslow argued that Townley’s evident and very public lack of remorse before witnesses at the scene was itself a significant symptom of insanity. When interviewed, Townley maintained that he had done the right thing as he believed that Miss Goodwin was effectively his property and that by killing her he was reclaiming that property and no others had the right to judge him. He also believed that he was the victim of a conspiracy, with her relatives seeking to undermine the proposed marriage. Well aware that by this time the court’s attention would be focused on the question of whether Townley understood right from wrong, Winslow had to concede that Townley ‘knew he had done a thing contrary to law’, but that at interview he had found that his ‘moral sense’ was ‘more vitiated than in any man I ever saw’:

He seemed incapable of reasoning upon any moral question that I brought before him. And he made the unaccountable assertion that he was not responsible to God or to man … He clenched his fists and his eyes started from his head, and as I thought he was going to have a paroxysm of maniacal fury – he said ‘I have done no murder, I am not a murderer’; I thought it would be unsafe to continue the investigation and I stopped.^11^

Winslow’s defence of Townley resembled that used in the cases of Oxford and M’Naghten (at whose trial Winslow had given expert evidence). The lack of remorse or even fear experienced in the shadow of the gallows was used as evidence of innocence. The judge, despite having some sympathy for Townley’s suffering such ‘agony of mind’ due to his heartbreak, was also very clear in his summing up that the judgment on insanity had to be made within the strictures of the M’Naghten rules. If Townley knew that what he did was likely to cause death and that he did it for that purpose, and if he knew ‘that in doing it he was doing what the law of God declares to be a bad act, a wrong act, contrary to the sixth commandment’, then he should be found guilty.^12^ Having been so strongly directed, it was perhaps not surprising that the jury took only five minutes to confirm the guilty verdict. The press reaction was more or less uniformly supportive of the verdict and celebrated the defeat of Winslow. A leader in *The Telegraph* was typical in taking a fairly withering tone:

The evidence of Dr Forbes Winslow is a social fact of some magnitude. That gentleman enjoys a high reputation, and his opinions are always heard with deference and respect. As long as they are expressed only in books or magazines – suggested simply as theories and hypotheses – they can do, if not good, at any rate little harm. But when the doctor enters the court of justice … it becomes absolutely essential to scrutinise his statements with greatest care.^13^

The leader was in little doubt as to the conclusion of such scrutiny of Winslow’s evidence; ‘his diagnosis was feeble and imperfect’ and had his conclusion been accepted ‘no-one would have been safe’. A comment piece from the *Liverpool Daily Mercury* was less polite and, under the title ‘The Mad Doctors Again’, it condemned Winslow’s attempt to suggest that Townley’s callous attack, combined with his lack of remorse and insistence that he had done right was evidence of insanity: ‘We are not aware that the conscience and common sense of mankind were ever more flagrantly outraged than by the respectable professor of medical science who gravely asks mankind to accept this revolting paradox’.^14^

All this might have been bad enough publicity for the new profession, but events prolonged and intensified the agony. Despite such public approval for the verdict, the trial judge, Baron Martin, seemed to waiver in the following days. He wrote immediately to the Home Secretary to suggest that caution might be needed regarding the planned execution, given Winslow’s claim that Townley was insane when he interviewed him. The Home Secretary seemed moved by this caution and requested that Townley be examined by the local Lunacy Commissioners. There was immediate outrage about this decision, for example: ‘… we have no hesitation in saying that the decision of the Home Office, unaccompanied by the publication of the evidence which can alone justify it, is about the severest blow that has been dealt in our time to the administration of justice.’^15^ The Lunacy Commissioners’ ambivalent conclusion summed up the problem rather than solving it. They found that Townley was not of ‘sound mind’ since he had an ‘extraordinarily perverted moral sense’ and insanity appeared to be in the family. However, they also agreed that he was still criminally responsible within ‘the law as laid down by Mr Baron Martin’ (Anon., 1864: 52). Meanwhile, a campaign, partly funded by Townley’s own wealthy family, pressed for clemency. Lawyers were employed to argue, with the help of paid medical witnesses, that Townley was insane. A series of petitions were raised^16^ and nine of the jurors from the trial wrote to the Home Secretary requesting clemency.^17^ The Home Secretary then stepped in to halt the execution and ordered that Townley should be sent to an asylum for further examination, so he was thus taken by train to ‘Bethlem’ Hospital in London on the 5 January 1864. Judging by the response of the press, there was considerable public outrage at this turn of events; for example: ‘Gentility shuddered at the idea of seeing “one of us” hanged’, and Townley’s affluent family had ‘spared neither gold nor exertion in his behalf’. The most damaging claim for psychiatry was that ‘Medical evidence … was bought in hard cash’.^18^ Another newspaper, having spelt out how straightforward the prosecution case had been, since Townley had proudly claimed responsibility for killing Miss Goodwin, was more specific in aiming fire at the new profession and at Winslow himself:

When in such circumstances, the worst comes to the worst, there are, thank goodness, the ‘mad doctors’. Accordingly, the attempt was made to prove Townley insane. With a felicitous ease only known to theorists, Dr Forbes Winslow proceeded to his demonstrations … Here is something like the Doctor’s allegation: - If, he says, a man kills another under the belief that he is responsible to the laws of neither God nor man, there is no murder, because a man who holds these opinions is insane.^19^

The conclusion of the examination at Bethlem Hospital was that Townley was of sound mind (Walker, 1968: 208) and thus he was transferred back to Pentonville prison with a sentence commuted to one of a lifetime of hard labour. Here, Townley himself ‘put an end to the tragic-comedy’ (p. 208). After singing two verses of ‘Abide with me’ in the prison chapel, he threw himself to his death over the balcony. This was, however, not quite end of the story as the jury at the Coroner’s inquest assumed him to be ‘morally insane’ and returned the verdict that he killed himself ‘whilst in an unsound state of mind’.^20^

## The professional response and the return to biology

The response of the new profession to this controversy was immediate. A special 47-page pamphlet by ‘the editors’ of *The Journal of Mental Science* was quickly rushed to press in January 1864, even before the sorry story had reached its final chapter at the coroner’s court. The pamphlet consisted chiefly of a review of the facts of the case and a sketch of the possible diagnoses that might have been applied to Townley. Three categories were described: (1) Monomania or Partial Intellectual Insanity; (2) Moral Insanity; and (3) Impulsive or Instinctive Insanity. The rhetorical question is asked: ‘What form of insanity, then, did Dr Winslow attribute it to?’ (Editors of JMC, 1864: 36–7). The answer suggests that such a question is ‘impossible to answer’ because ‘Townley’s insanity, as described by that psychologist was a medley, a scientific patchwork, ingeniously constructed, boldly devised, striking in appearance, but really a scientific incoherency – a mixture of incompatibles’ (p. 36). This criticism is a little disingenuous, as the defences of Oxford and M’Naghten certainly used a similar array of language and concepts. Despite the stated uncertainty about the diagnosis used in the trial, it is notable that more space is given to the category of ‘moral insanity’, and it is the only category in which parallels were noted between the concept and Winslow’s defence in the court. It is argued that this diagnosis was not appropriate since, although Townley’s mind might have reasonably been described as morally depraved, there was no sign of any form of disease before the act itself. The pamphlet concludes by arguing that courts ought to take medical testimony seriously, and that medical experts should be paid by the state to investigate. Had Townely been examined by ‘impartial and skilful physicians’, they would have failed to find any disease that might have caused the crime (p. 45) – the implication being that the problem was Winslow’s eagerness to provide a testimony, for which he was paid.

Following up on the pamphlet, a considerable section of the first volume of *The Journal of Mental Science*^21^ published in 1864 was devoted to discussion of the case, in particular a piece by John Hitchman, Physician Superintendent of Derby County Asylum, who had been invited by Townley’s defence team to interview him. Hitchman’s detailed report of a two-hour interview gives a similar impression to that given by Winslow in the trial. Hitchman concludes that, despite Townley’s apparent lack of moral compass and indifference to his own fate, he could not support the view that Townley was ‘currently’ insane. Hitchman had indeed written to Townley’s defence attorney, Mr Leech, telling him that he could not argue for the defence of insanity in court. While noting that Townley had ‘a feeble intellect associated with strong emotions’ and a ‘hereditary predisposition to mental disease’, which could mean that he might in the future lose his sanity, he was currently ‘a rational and responsible person’ (Hitchman, 1864: 28). Hitchman’s report concludes: ‘I allege that Mr Townley is not now insane, in the legal sense of that term, because he is under no hallucination; because absurd as are his dogmas, in reference to man’s responsibility … they are theories entertained by hundreds of persons who are capable of all duties of social life’^22^ (p. 28).

It was perhaps no wonder that the profession was so keen to distance itself from Winslow’s defence. The Townley affair had an immediate impact on press reports of other trials. One, under the heading ‘Another Result of Townley’s Reprieve’, reported on the case of Ralph Wibberley, who was charged with threatening to cut off his wife’s head and set fire to her. She claimed that he had been influenced by the Townley verdict, as he told her: ‘Now they have let that poor fellow off, there is no law for me; I will have my revenge’, and he had constantly assaulted her since then.^23^ In the following year, 1865, the case of the Ramsgate and Holborn murderer Stephen Forwood (also known as Southey) held a certain public fascination; he had murdered his wife, girlfriend and three children. Despite the very obvious eccentricities^24^ of the accused and his conduct in court, the accounts of the trial suggest that no serious medical case was put forward as to insanity. In 1867 the notorious murder of eight-year-old Fanny Adams by 24-year-old Frederick Baker provoked no serious debate about the sanity of the killer who had abducted, killed and dismembered the little girl during his working day as an office clerk. Although he had blood on his clothes and had written in his own diary ‘Killed a little girl; it was fine and hot’, he still claimed innocence and made no attempt to escape. Although the issue of insanity, and ‘homicidal mania’ in particular, was raised in the court, it was not seriously supported. The judge argued that the magnitude of the crime should never be taken as evidence of insanity. An article in *The Journal of Mental Science* made no serious effort to contradict this point: ‘It is not possible, we fear, to call him actually insane, unless we are content to give up all exact notions of insanity.’ Nevertheless, as the comment continued, there were grounds to doubt his sanity:

… there is little doubt that had his life been prolonged, he would have become insane. The evidence at that the trial showed that a near relative of his father was in confinement suffering from homicidal mania and that his father had an attack of acute mania. Moreover, it was proved in evidence by independent witnesses that he himself had been unlike other people, that he had been prone to weep frequently without evident reason, that he had exhibited singular caprices of conduct, and that it had been necessary to watch him from the fear that he might commit suicide. (Anon., 1868)

If Baker had been tried before the M’Naghten rules were fully accepted, the course of his trial may well have been very different; at the very least, the issue of insanity would probably have been raised far more prominently. Indeed, some years later Henry Maudsley himself invoked ideas of homicidal insanity (as a form of monomania or affective insanity) in order to explain Baker’s behaviour: ‘the impulsive character of the crime, the quiet and determined ferocity of it, the savage mutilation, his equanimity immediately afterwards, and his complete indifference to his fate – all these indicated an insane organisation’ (Maudsley, 1874: 163). The absence of ‘psychiatric’ testimony in this trial, in the shadow of the Townley affair, is striking. While versions of the insanity defence were used in the late nineteenth century (a number of interesting cases are discussed by Wiener 1999, for example), the specific defences of ‘moral insanity’ or ‘monomania’ were not used.

The conclusions of the pamphlet produced by the Editors of *The Journal of Mental Science* (1864) were that the legal maxims of responsibility reinforced by the M’Naghten ‘rules’ were too narrow and that expert witnesses in insanity should be appointed by the court rather than either the defence or prosecution.^25^ While these proposals were not taken up, the new experts in insanity were still keen to engage with the law and criminality, but they abandoned their claim to be able to detect hidden forms of dangerous mental disorder in particular individuals. Instead, they turned to the examination and categorization of the mass of the so-called criminal classes and to distinctly biological theories of mental disorder. Thomas Laycock, as President-elect of the Medico-Psychological Association (and Professor of Medicine and Medical Psychology at the University of Edinburgh) made this shift entirely clear in an address given to the Association (Laycock, 1868). He noted the great difficulties in trying to establish the presence of insanity in those court cases where the defendant knew that murder was wrong but who might still be driven to such acts. Laycock then turned his attention to discussion of the larger problem of the ‘*classes dangereuses*’ (which he translated as ‘known to the police’) and the even larger groups of ‘incorrigible vagabonds, drunkards, mendicants’ who in their tens of thousands were:

so constituted corporally that they possess no self-control beyond that of an ordinary brute animal – nay less than a well-bred horse or dog. They are, for the most part, immoral imbeciles, so that however frequently they may have been subjected to prison or other discipline, the moment they are set free, they resume their vicious and criminal course … They are all the mere weeds of society, but, like weeds they multiply their kind, and thus continually keep up the breed. (Laycock, 1868: 342–3)

Invoking the language of horticulture, Laycock directs the profession towards eugenics as the means needed to be found to control ‘their personal liberty during the fertile period of life’ (p. 344). He was far from being a lone voice in arguing for a more organic perspective on mental disorder. Well before the end of the nineteenth century, the shift of the profession of psychiatry towards biological speculation was very clear (Maudsely, 1868), including the branches of the profession concerned with criminality (Davie, 2010; Thomson, 1869, 1870).

## Discussion

The story of ‘moral insanity’ (and related disorders) has had remarkable, but often unacknowledged, influence on the shape of the psychiatric profession (Rafter, 2004), particularly in Britain. The idea that there might be very particular disorders which could explain someone’s violent behaviour, and be detected through examination by experts, was particularly beguiling in the early years of the profession as it fought for public and professional recognition. The trial of Daniel M’Naghten triggered the downfall of moral insanity in the courts. This is arguably one of the most significant trials in legal history as it led to the formation of the so-called M’Naghten rules which tightened the criteria circumscribing the insanity defence. It took a couple of decades for the full ramifications of the ruling to become clear to the new profession, and the public press-fuelled disparagement of concepts of moral insanity, particularly during the trial of George Victor Townley, pressured psychiatrists to abandon this territory in the courts.

Still concerned, however, with making a contribution to criminal justice, psychiatry turned to the prison population to claim expertise in the categorization and management of risk. Here, theorization became thoroughly enmeshed with theories of degeneracy and wider cultural anxieties about the downfall of western culture and its population (Pick, 1989). While the profession of psychiatry became embroiled in organic theorization, the idea of more subtle kinds of insanity that might only exist and be explored in the psychological realm was taken up enthusiastically in the wider culture, particularly in the world of fictional literature which was assuming growing significance in the nineteenth century (Jones, 2016). By the end of that century, the wider cultural acceptance and discussion of the human psyche as consisting of hidden depths and contrary impulses proved crucial to the other great development of practice and thinking – psychoanalysis.

This story helps us to understand more of the contradictory forces that have shaped psychiatry. The profession has needed the claim for expertise in matters of criminality, but has also been very sensitive to public opinion on those claims. Close attention to the early decades of the profession’s progress in the first half of the nineteenth century shows that there was strong interest in psychological modes of expertise. These are often forgotten, as the profession retreated, under the force of public pressure, from making courtroom claims about moral insanity.

## Notes

1. The organization received a royal charter in 1926 and thus became the Royal Medico Psychological Association; it was not until 1971 that it became a medical Royal College.

2. I am grateful for the availability online of the ‘Proceedings of the Old Bailey 1674–1913’ (hereafter OBP) that have enabled the analysis of these cases; see: www.oldbaileyonline.org, version 7.0, 26 January 2015. For discussion of this resource, see Shoemaker, 2008.

3. OBP, July 1840, trial of Edward Oxford (t18400706-1877).

4. OBP, February 1843, trial of Daniel M’Naughten (t18430227-874).

5. Sir Alexander Morison, visiting physician at Bethlem,William Hutchinson, physician at the Royal Lunatic Asylumn in Glasgow, Mr William McClure, a surgeon, and Dr Sutherland and Dr Bight were also present.

6. See note 4.

7. See note 4.

8. See note 4.

9. *The Standard*, 6 Mar. 1843, original italics.

10. *The Morning Post* (London), 14 Mar. 1843 (issue 22512): 2.

11. *Derby Mercury*, 16 Mar. 1863: 3–4.

12. See note 11.

13. *The Telegraph* leader, quoted in *Derby Mercury* 16 Dec. 1863: 3–4.

14. *Liverpool Mercury*, 4 Jan. 1864 (issue 4962): 1.

15. See note 14.

16. Petitions were signed by 963 males and 584 females from Derby, 371 from Wigwell, 140 from Burton-on-Trent and the very large figure of 16,709 from Townley’s home town of Manchester. Another petition, raised through immediate friends, had 7938 signatures (3251 of them women); Anon., 1864: 39.

17. They wrote: ‘We believe the verdict to be perfectly legal and just, but owing to an absence of premeditation on the part of the prisoner, his previous good character, and the state of his mind as described by the medical evidence, we venture to suggest that the extreme penalty of the law should not be carried into effect without further consideration being given to the case.’; Anon., 1864: 39.

18. *Lloyd’s Weekly Newspaper* (London), 14 Feb. 1864 (issue 1108).

19. *The Caledonian Mercury*, 25 Dec. 1863.

20. *Suicide of Victor Townley, who was found guilty and sentenced to death for the murder of Miss Goodwin at Manchester*. [London]: Disley [*c*. 186-?]; accessed (23 Feb. 2017) at: http://pds.lib.harvard.edu/pds/view/4788255; also: Suicide of George Victor Townley. *Wellington Independent* XX (18 May 1865, issue 2207).

21. Formerly *The Asylum Journal*, produced by the Association of Medical Officers of Asylums.

22. This is reference to godless views of the ‘necessitarians’, championed by Percy Shelley.

23. *The Blackburn Standard*, 24 Feb. 1864.

24. As described by *The Spectator*, 19 Aug. 1865: 9.

25. Dr Bucknill is quoted, to give a flavour of the perception that medical witnesses could be bought: ‘An array of medical men are marshalled by the attorneys on each side according to their preconceived opinions of the case. These medical witnesses may usually be divided into two classes – those who know something of the prisoner and nothing of insanity, and those who know something about insanity and nothing of the prisoner. They generally succeed in neutralizing each other’s evidence, and in bringing the medical profession into contempt, at least among lawyers.’ ; Editors of JMC, 1864: 46.

## References

[bibr1-0957154X17702316] Anonymous (1800) The Trial of James Hadfield for High Treason. The Whole of the Evidence. London: W.I. Clement.

[bibr2-0957154X17702316] Anonymous (1827) Homicide-suicide-infanticide: the case of Harriet [*sic*] Cornier. Medico-Chirugical Review: Quarterly Periscope 6(12): 482–487.

[bibr3-0957154X17702316] Anonymous (1853) Prospectus. The Asylum Journal 1: 2.

[bibr4-0957154X17702316] Anonymous (1864) The Trial and Respite of George Victor Townley for Wilful Murder. With Original Documents and Correspondence. Derby: Bemrose and Sons.

[bibr5-0957154X17702316] Anonymous (1868) Comment. Journal of Mental Science XIII: 38.

[bibr6-0957154X17702316] Augstein HF (1996) JC Prichard’s concept of moral insanity – a medical theory of the corruption of human nature. Medical History 40: 311–343. 8757717 10.1017/s0025727300061329 PMC1037128

[bibr7-0957154X17702316] Berrios GE (1993) European views on personality disorders: a conceptual history. Comprehensive Psychiatry 34(1): 14–30. 8425387 10.1016/0010-440x(93)90031-x

[bibr8-0957154X17702316] Boime A (1991) Portraying monomaniacs to service the alienist’s monomania: Gericault and Georget. Oxford Art Journal 14(1): 79–91.

[bibr9-0957154X17702316] Bracton H (*c*1250) De Legibus et Consuetudinibus Angliæ (On the Laws and Customs of England); accessed (23 Feb. 2017) at: http://bracton.law.harvard.edu/

[bibr10-0957154X17702316] Burns LC (1954) A forgotten psychiatrist – Baron Ernst von Feuchtersleben. Proceedings of the Royal Society of Medicine 47(3): 190–194. 13155507 10.1177/003591575404700312 PMC1918589

[bibr11-0957154X17702316] Bynum TF (1964) Rationales for therapy in British psychiatry: 1780–1835. Medical History 18: 317–344. 10.1017/s0025727300019761 PMC1081592 4618306

[bibr12-0957154X17702316] Castel R (1988) The Regulation of Madness: The Origins of Incarceration in France, trans. Hall WD Cambridge: Polity Press; originally published in 1977 as *L’Ordre psychiatrique: L’âge d’or de l’aliénisme*.

[bibr13-0957154X17702316] Davie N (2010) The impact of criminal anthropology in Britain (1880–1918). Criminocorpus, revue hypermédia [En ligne], Histoire de la criminologie, 4. L’anthropologie criminelle en Europe; accessed (23 Feb. 2017) at: http://criminocorpus.revues.org/319; DOI:

[bibr14-0957154X17702316] Delasiauve [no initial] (1853) On monomania, in a psychological and legal point of view; abridged by J.T. Aldridge from *Annales Medico-Psychologiques* July 1853. The Asylum Journal 1: 8–10.

[bibr15-0957154X17702316] During S (1988) The strange case of monomania: patriarchy in literature, murder in Middlemarch, drowning in Daniel Deronda. Representations 23: 86–104.

[bibr16-0957154X17702316] Editors of *The Journal of Mental Science* (1864) Insanity and Crime: A Medico-legal Commentary on the Case of George Victor Townley. London: John Churchill and Sons.

[bibr17-0957154X17702316] Eigen JP (1991) Delusion in the courtroom: the role of partial insanity in early forensic testimony. Medical History 35: 25–49. 2008121 10.1017/s0025727300053114 PMC1036268

[bibr18-0957154X17702316] Eigen JP Andoll G (1986) From mad-doctor to forensic witness: the evolution of early English court psychiartry. International Journal of Law and Psychiatry 9: 159–169. 3542855 10.1016/0160-2527(86)90044-0

[bibr19-0957154X17702316] Esquirol J-ED (1819) Monomanie. In: Dictionnaire des sciences médicales, 34 Paris: Panckoucke, 114–125.

[bibr20-0957154X17702316] Foucault M (1967) Madness and Civilization: A History of Insanity in the Age of Reason, trans. Howard R.; London: Tavistock Publications; originally published in 1961 as *Histoire de la folie à l’âge classique*.

[bibr21-0957154X17702316] Foucault M (2003) Abnormal: Lectures at the Collège de France 1974–1975, trans. Burchell G London: Verso; originally published in 1999 as *Les Anormaux Cours aux Collège de France*.

[bibr22-0957154X17702316] Georget JE (1826) Discussion medico-légal sur la folie ou alienation mentale, suivie de l’examen du process criminal d’Henriette Cornier. Paris: Migneret.

[bibr23-0957154X17702316] Goldstein J (1987) Console and Classify: The French Psychiatric Profession in the Nineteenth Century. Cambridge: Cambridge University Press.

[bibr24-0957154X17702316] Goldstein J (1998) Professional knowledge and professional self-interest: the rise and fall of monomania in 19th-century France. International Journal of Law and Psychiatry 21(4): 385–396. 9870179 10.1016/s0160-2527(98)00029-6

[bibr25-0957154X17702316] Hale M (1736) History of the Pleas of the Crown [written *c*1680]. London: Giles.

[bibr26-0957154X17702316] Hansen LA (1998) Metaphors of mind and society: the origins of German psychiatry in the revolutionary era. Isis 89(3): 387–409. 10.1086/384071 9798338

[bibr27-0957154X17702316] Hitchman J (1864) An interview with George Victor Townley, and reflections thereon. Journal of Mental Science 10: 21–34.

[bibr28-0957154X17702316] Jones DW (2016) Disordered Personalities and Crime: An Analysis of the History of Moral Insanity. Abingdon: Routledge.

[bibr29-0957154X17702316] Laycock T (1868) Suggestions for rendering medico-mental science available to the better administration of justice and the more effectual prevention of lunacy and crime. Journal of Mental Science 15(67): 334–345.

[bibr30-0957154X17702316] Loughnan A (2007) ‘Manifest madness’: towards a new understanding of the insanity defence. Modern Law Review 70(3): 379–401.

[bibr31-0957154X17702316] Loughnan A (2012) Manifest Madness: Mental Incapacity in Criminal Law. Oxford: Oxford University Press.

[bibr32-0957154X17702316] Marková IS Berrios GE (2012) Epistemology of psychiatry. Psychopathology 45(4): 220–227. 22627668 10.1159/000331599

[bibr33-0957154X17702316] Maudsley H (1868) Illustrations of a variety of insanity. Journal of Mental Science 14(66): 149–162.

[bibr34-0957154X17702316] Maudsley H (1874) Responsibility in Mental Disease. Appleton: New York.

[bibr35-0957154X17702316] Moran R (1985) The origin of insanity as a special verdict: the trial for treason of James Hadfield (1800). Law & Society Review 19(3): 487–519. 11617589

[bibr36-0957154X17702316] Parkin A (1975) Feuchtersleben: a forgotten forerunner to Freud. Canadian Psychiatriac Association Journal 20(6): 477–481. 10.1177/070674377502000609 1104133

[bibr37-0957154X17702316] Pick D (1989) Faces of Degeneration: A European Disorder, c.1848–1918. Cambridge: Cambridge University Press.

[bibr38-0957154X17702316] Pinel P (1806) A Treatise on Insanity: In which are Contained the Principles of a New and More Practical Nosology of Maniacal Disorders Than Has Yet Been Offered to the Public, trans. Davis DD London: Caddell and Davies; originally published in French in 1801.

[bibr39-0957154X17702316] Porter R (2002) Madness: A Brief History. Oxford: Oxford University Press.

[bibr40-0957154X17702316] Prichard JC (1833) Insanity. In: Forbes J Tweedie A Conolly J (eds) The Cyclopædia of Practical Medicine, Vol. 2 London: Sherwood, Gilbert, and Piper, 824–875.

[bibr41-0957154X17702316] Prichard JC (1835) A Treatise on Insanity and Other Disorders Affecting the Mind. London: Sherwood, Gilbert and Piper.

[bibr42-0957154X17702316] Rafter N (2004) The unrepentant horse-slasher: moral insanity and the origins of criminological thought. Criminology 42(4): 979–1008.

[bibr43-0957154X17702316] Richards RJ (1998) Rhapsodies on a cat-piano, or Johann Christian Reil and the found psychiatry. Critical Inquiry 24(3): 700–736.

[bibr44-0957154X17702316] Rimke H Hunt A (2002) From sinners to de-generates: the medicalization of morality in the 19th century. History of the Human Sciences 15(1): 59–88. 21038728 10.1177/0952695102015001073

[bibr45-0957154X17702316] Rousseau GS (1969) Science and the discovery of the imagination in Enlightened England. Eighteenth-Century Studies 3(1, Special Issue: The Eighteenth-Century Imagination): 108–135. 19950449

[bibr46-0957154X17702316] Scull A (1979a) Museums of Madness: The Social Organization of Insanity in Nineteenth Century England. London: Allen Lane.

[bibr47-0957154X17702316] Scull A (1979b) Moral treatment reconsidered: some sociological comments on an episode in the history of British psychiatry. Psychological Medicine 9: 421–428. 384444 10.1017/s0033291700031962

[bibr48-0957154X17702316] Scull A (1984) A brilliant career? John Connolly and Victorian psychiatry. Victorian Studies 27(2): 203–221. 11621271

[bibr49-0957154X17702316] Shoemaker RB (2008) The Old Bailey proceedings and the representation of crime and criminal justice in eighteenth-century London. Journal of British Studies 47: 559–580.

[bibr50-0957154X17702316] Smith R (1981) Trial by Medicine: Insanity and Responsibility in Victorian Trials. Edinburgh: Edinburgh University Press.

[bibr51-0957154X17702316] Thomson JB (1869) The hereditary nature of crime. Journal of Mental Science 36: 487–498.

[bibr52-0957154X17702316] Thomson JB (1870) The psychology of criminals. Journal of Mental Science 39: 321–350.

[bibr53-0957154X17702316] Tuke S (1813) Description of The Retreat, an Institution near York. York: W. Alexander. 20262289

[bibr54-0957154X17702316] Von Feuchtersleben E (1847) The Principles of Medical Psychology, trans. Lloyd E London: Sydenham Society; originally published in 1845 as *Lehrbuch Der Ärztlichen Seelenkunde: Als Skizze Zu Vorträgen*.

[bibr55-0957154X17702316] Walker N (1968) Crime and Insanity in England. Volume 1: The Historical Perspectives. Edinburgh: Edinburgh University Press.

[bibr56-0957154X17702316] Werlinder H (1978) Psychopathy: A History of the Concepts Analysis of the Origin and Development of a Family of Concepts in Psychopathology. Uppsala: Acta Universitatis Upsaliensis.

[bibr57-0957154X17702316] Wiener MJ (1999) Judges vs. jurors: courtroom tensions in murder trials and the law of criminal responsibility in nineteenth-century England. Law and History Review 17(3): 467–506.

[bibr58-0957154X17702316] Winslow FH (1843) The Plea of Insanity in Criminal Cases. London: H. Renshaw.

